# Southern Medical College Opening Exercises

**Published:** 1887-11

**Authors:** 


### SOUTHERN MEDICAL COLLEGE—OPENING EXERCISES.

We clip from the *Atlanta Constitution* the following report of the opening of the above popular institution:

"The opening exercises of the Southern Medical College occurred yesterday morning at 11 o'clock in the Chapel of the College, there being a number of citizens and a large medical class in attendance.

"The introductory address was made by Dr. R. C. Word, Professor of Physiology. The doctor's address was an admirable one. It embraced a retrospective view of the last forty years, and the speaker passed in rapid review the manifold advances made in the science of medicine during this period Thompsonianism, Eclecticism, and Homœopathy were described, and contrasted with the regular profession. Ether as an anæsthetic was commended, and its value was shown. Dr. Word claimed that it was used first in the South, and the first experiment ever made with it was made in Georgia. Touching materia medica the speaker had much to say. His remarks regarding surgery were instructive and even startling. He told of the wonders of this art; described some of the marvelous cures effected by the skillful use of the knife; related how a man's intestines, five feet in length, had been successfully removed, and how, marvelous to tell, the patient had recovered.

"He spoke of the doctors during the war, and claimed that, despite the deficiency of drugs and surgical appliances in the South, the Southern Surgeons compared favorably with those of the North in all departments. He mentioned their great skill and ingenuity in improvising surgical devices, and in the development and use of idigenous plants and medicines. He spoke of the professional courtesy and kindness that existed between the surgeons of the opposing forces, when through the fortunes of war they were thrown together or fell into each other's hands. He showed how great were the sacrifices of medical men during the war—whether in the army or at home—those who remained at home being scorned by the soldiery, though assisting in taking care of soldiers' families and ministering to the needs of the poor.

"The great blow inflicted upon the emoluments of the practice by reason of the abolition of slavery and the ruin of Southern planters, was mentioned 'Of the Southern planters before the war, especially those in the cotton belt,' he said, 'they were the best and noblest of all the types of men ever known to the world. Take an example of a well-to-do Southern planter in his elegant home. A cavalier in deportment, jealous of his honor, generous, frank and genial in disposition, free and liberal in the use of his money, hospitable almost to a fault, charitable to the poor, beloved by his slaves, to whom he was ever kind, he was in all respects a high-toned gentleman and a true man.'

"Of the society in that day he remarked: "Whatever may be said of domestic slavery in the South, it has often been said, and I believe truthfully, that society as it then existed, and as illustrated in our own Middle Georgia, constituted the grandest type of civilization ever known in the annals of history—no infidelity, no fanaticisms, no conflict between labor and capital, no communism, no socialism, no cravings of hunger, no shiverings in the wintry blast for want of fuel, the laboring element being well cared for, cheerful, contented and happy. That slavery had abuses I do not deny, but so has the marriage relation, and all other institutions known to a wicked world, but viewed in the light of average results, or judged by the rule of the greatest good to the greatest number there has been no class of laborers in the world so happy—no society so free from demoralization as was found in the Old South, reviled, misrepresented and persecuted though she was.'

"'I have referred to the war,' continued Dr. Word, 'because I believe it a duty of all Southern teachers, writers and public men, now and then to instruct our young people in the facts of the war, and thus let the truth be handed down to coming generations, that they may know our side of the question as we feel it; and as we endured it. This should be done, not to prejudice our youth against the North, but rather that the whole truth may be known, and in the hope that a free expression of our feelings, and a full understanding of each other, will bring us together and restore harmony to the sections.

"Many of the books in our schools are untrue to history and calculated to give our children, both of the present and coming generations, false and disparaging views of the patriotism of their fathers and of the cause for which they so nobly struggled.

"The gradual unfolding of the real facts of the so-called rebellion, the true character of our people and the great truths to which I have referred, are beginning to impress the minds of thinking and unprejudiced men, and a sense of returning justice to our section is manifest everywhere, even in the Northern section of our Union.

"After urging the students to be punctual, sober, studious, moral and upright, and disclaiming any reflection upon students from the North who were present, he closed with this remark, addressed to such:

"'The New South is a part of the American Union. She welcomes you here, she rejoices in the welfare of the whole country, and all that impartial history shall record of our achievments in the past, and all that we shall accomplish in the future will be yours to enjoy as the common heritage of a reunited and prosperous people.'

"Dr. Word's address was followed by some remarks by Dr. T. S. Powell. He complimented the speaker upon his faithfulness and eloquent discussion of his subject, "Then and Now," and the truthfulness of the retrospect he had given of the advances made in the last forty years of the profession. The superiority of the best class of medical colleges of the present day over the colleges of the 'then,' to which the speaker referred, is true. He mentioned in this connection the advantages offered by the Southern Medical College, and gave a forcible sketch of the great difficulties he had overcome in establishing this great enterprise.

"He said that all that had been promised to the profession had been fulfilled, the college building being complete and equipped in all departments, including a hospital. In this important feature he was greatly indebted to the good ladies of the city, who are still at work in behalf of this noble enterprise. Upon the ladies the doctor grew eloquent, as he always does, concluding with the remark: "God bless the women of Atlanta; God bless the women of the world, the guardian angels of man, the inspirers of noble character, the builders of happy homes, the evangels of the world.'

"In the conclusion of his remarks he presented to the audience the Dental Faculty, composed of seven gentlemen of the city, whom he highly complimented for their qualifications for their respective places. They had been selected, just as every young man should select his wife, 'on their merits.' The doctor's frequent witicisms and happy hits imparted a pleasing and happy flavor. He introduced Dr. Holland, who spoke in behalf of the dental department. Dr. Holland was very facetious in the opening remarks of his address. He paid a high compliment to the members of the medical faculty, and especially to the President, Dr. T. S. Powell, alluding in strong terms to his indomitable energy and his great wisdom in the prosecution of great enterprises. He was enthusiastic about the good which Powell had accomplished for the profession in the South and for Atlanta. A due appreciation, he thought, of his efforts would bring a sense of obligation, especially on the part of our citizens, and he would only have to ask what he wanted for the accomplishment of his enterprises and it would be granted by our people, and the institution over which he presides would be endowed by some great benevolent heart. The concluding remarks of Dr. Holland were instructive and admonitory to the students, being replete with sound sense and good advice.

The exercises were heartily enjoyed by all present.

The prospects of the college are brighter than ever, and there will be a very large attendance this session.

